# Detection of Hepatitis B Surface Antigen among Febrile Patients in Ankpa, Kogi State, Nigeria

**DOI:** 10.1155/2020/5136785

**Published:** 2020-02-11

**Authors:** Cornelius Arome Omatola, Bernard Anyebe Onoja, Joseph Agama

**Affiliations:** ^1^Department of Microbiology, Kogi State University, Anyigba P. M. B. 1008, Kogi State, Nigeria; ^2^Department of Virology, University College Hospital, Ibadan, Oyo State, Nigeria

## Abstract

**Background:**

Hepatitis B virus (HBV) infection has become a significant public health problem in developing countries, and the high rate of morbidity and mortality from acute and chronic infections is worrisome. Therefore, this study determined the prevalence of HBV and associated risk factors in Ankpa, Kogi State, Nigeria. *Materials and Methods*. Sera randomly collected from 200 participants in three public hospitals in Ankpa were screened for HBsAg using commercially available HBsAg rapid test kit (Swe-Care (R), China). Structured questionnaires were used to obtain sociodemographic details and history of exposure to risk factors.

**Results:**

Seventeen (8.5%) of the 200 patients were positive for HBsAg. Males had higher prevalence (10.89%) than females (6.06%). The age group with the highest rate of infection was 24–44 years. Patient's occupation and marital status were significantly higher in relation to HBsAg seropositivity. Risks of HBV infection in Ankpa are sharing of sharp objects (OR = 11.62, 95% CI, 3.59–37.59), multiple sexual partners (OR = 3.39, 95% CI, 1.23–9.38), blood transfusion (OR = 13.74, 95% CI, 4.22–44.71), surgeries (OR = 3.02, 95% CI, 1.03–8.83), alcoholism (OR = 6.94, 95% CI, 2.32–20.75), mouth-to-mouth kissing (*p*=0.001), and contact with HBV patient (OR = 4.14, 95% CI, 1.01–17.06). People without prior knowledge of HBV infection were more infected.

**Conclusion:**

This study reaffirms the endemicity of HBV in a part of sub-Saharan African country. Public health practitioners should focus attention on apparently healthy patients in developing countries. We suggest inclusion of HBsAg screening for patients coming for routine hospital care.

## 1. Introduction

Hepatitis caused by hepatitis B virus (HBV) is a life-threatening liver infection that causes cirrhosis and hepatocellular carcinoma [1]. Globally, more than 2 billion people are infected with HBV [2]. About 350 million people are chronic HBV carriers [3], with over 8% in sub-Saharan Africa [4]. In sub-Saharan Africa, a high rate of asymptomatic HBV infection exists and poses significant health risk for close family contacts and public health workers.

The World Health Organization categorizes low prevalence as ≤1.9%, moderate prevalence as 2–7.9%, and high prevalence as >8% HBsAg positivity [5]. HBV has been reported in different populations in various parts of Nigeria [6–11]. It is a serious public health problem affecting all ages. The likelihood of becoming a chronic carrier is higher for infection at infancy and early childhood [12]. About 10% of infected adults, 90% of infants infected at birth, and 50% of children infected in the age group of 1–5 years are at risk of developing chronic hepatitis [3]. There is increased risk for about 25% of patients who will later suffer from liver cirrhosis and/or liver cancer, if not medically managed [3]. Progression of liver disease to hepatocellular carcinoma was recently ranked as the third frequent cause of death in HBV endemic countries, constituting a heavy burden for low-income countries [13, 14].

HBV infection is referred to as a silent killer because majority of carriers are not aware they are carrying the virus and consequently fail to seek appropriate medical attention [15]. Early detection and prompt treatment is key to breaking transmission [16]. There is dearth of information on prevalence of HBV and associated risk factors in Ankpa, Kogi State, Nigeria. Therefore, we determined seroprevalence of HBsAg and predisposing risk factors among general outpatients attending clinics in Ankpa. We also assessed their knowledge about HBV infection and its prevention.

## 2. Materials and Methods

### 2.1. Study Area

The study was carried out in Ankpa, Kogi State, which is situated on latitude 7°14′-7°22′ N and longitude 7°31′-7°37′ E. It covers an area of 1,200 km^2^ with a population of 267,353. It lies on altitude 163 m above the sea level and has a heterogeneous population that is dominated by the Igala people.

### 2.2. Study Design/Population

A cross-sectional study was conducted from March to May 2016. Patients that presented with febrile illnesses were consecutively recruited by random sampling from the general outpatient departments of selected hospitals. Fever is an indication for querying infectious diseases. The stage of infection was not taken into account. Patients with established clinical conditions such as malaria, HIV/AIDS, cancer, sickle cell disease, and diabetes mellitus and patients already on course of chemotherapy or who had it in the last two weeks for treatment of an already diagnosed illnesses were excluded from participation in the study. Every third febrile patient who came in to visit the doctor during this period was recruited into the study. A total of 200 patients attending three major hospitals, namely, Living Hope, Amnesty, and Bethel hospitals participated in the study. Ethical approval was obtained from the ethical committee of each hospital in accordance with the Helsinki Code of Conduct for Biomedical Research involving human subjects. Informed consent was sought and obtained from each person before sample collection began. Sample size (*N*) was calculated using formula *N* = qZ*α*2pq/d2, where Z*α* = standard normal deviate set at 1.96, corresponding to 95% confidence level; *p* = proportion in the target population estimated to have a variable characteristic = 85% (0.85); *q* = 1−p = 15% (0.15); and *d* = degree of precision set at 0.05 (95% confidence interval). Therefore, *N* = 196, which was approximated to the nearest hundred as 200.

### 2.3. Sample Collection

Five milliliters of venous blood was aseptically collected into plain tubes from each consenting patient. Overall, 200 blood samples were collected. Sera were separated after centrifugation at 3,000 revolutions per minute for 5 minutes and stored at −20°C until tested. A structured questionnaire was used to collect demographic data and information on risk factors.

### 2.4. Detection of HBsAg

Serum from each participant was screened in a stepwise order using a HBsAg test kit (Swe-Care (R), China). It is an immunochromatographic and qualitative assay that detects HBsAg in human blood, with 99.9% sensitivity and 99.75% specificity. Each strip is precoated with monoclonal anti-HBs capture antibody. During testing, mouse monoclonal anti-HBs-colloid gold conjugate and samples move along the nitrocellulose membrane chromatographically to the test region (*T*) forming a visible band as antibody-antigen-antibody gold complex forms. Normally, both test and control lines are not visible before applying the sample. Control band is used as internal/procedural control. This should always appear if the procedure is performed accurately and reagents work properly. In interpretation of test result, only one band appears on the control region with no band on the test region for the negative test. This indicates no detectable HBsAg in serum. Positive result shows distinct bands on control and test regions, indicating that the specimen contains detectable amounts of HBsAg. An invalid test shows no visible band at all or only one band on the test region; this is an indication of a possible error in performing the test. Such test is repeated.

### 2.5. Data Analysis

Data generated were analyzed using Statistical Packages for Social Sciences (SPSS) 16.0 for Windows (Inc., Chicago, IL). Differences in proportion were compared using chi square. Level of statistical significance was set at *p* ≤ 0.05. Odds ratio was used to measure the risk factor independently associated with HBV transmission and computed by means of logistic regression at 95% confidence level.

## 3. Results

Of the 200 participants screened, 17 (8.5%) were positive for HBsAg. There were 101 males and 99 females (male-to-female ratio of 1.02 : 1.0). Gender-specific prevalence showed males had higher prevalence 11 (10.9%) than the females 6 (6.1%). However, there was no significant association between gender and HBV infection (*p* > 0.05). Age distribution of HBsAg showed a higher prevalence of 14 (15.4%) among those in the age group of 24–44 yrs, compared to those in the age group of 3–23 yrs, 45–65 yrs, and 66–86 yrs with 1 (1.4%), 2 (8.0%), and 0% prevalence rates, respectively. There was a statistically significant association between patient's age and HBsAg seropositivity (*p* = 0.01). Participants who were married had a higher prevalence of 13 (12.6%) while those who were single and divorced had 4 (4.1%) and 0% rates, respectively. There was a significant association between marital status and HBsAg (*p* = 0.03). Businessmen/women had higher HBV prevalence rates of 8 (28.57%) compared to those in the other occupations. Housewives had a prevalence of 5 (14.71%), farmers, 1 (14.29%), civil servants, 1 (3.45%), students, 2 (2.11%), and those who were unemployed (0%). There was a statistically significant association between occupation and HBsAg seropositivity (*p* = 0.01).  Table 1 depicts the frequency of potential risk factors reported by subjects with HBsAg seroprevalence rates and the OR estimated by univariate analysis. There was a statistical significant difference between HBsAg seropositivity and factors such as mouth-to-mouth kissing (*p* = 0.001), sharing of sharp objects (*p* = 0.01), multiple sexual behaviors (*p* = 0.014), alcoholism (*p* = 0.01), history of blood transfusion (*p* = 0.01), and surgeries and family history of HBV infection (*p* = 0.04).

## 4. Discussion

In this study, 8.5% of the patients who presented with fever as a symptom to the three major hospitals in Ankpa City were seropositive to HBsAg. In accordance with the World Health Organization classification of assessing severity of HBV infection in endemic countries, the rate observed in this study is regarded as high seroprevalence level of HBV infection. WHO in 2010, defines low prevalence to be <2%, moderate prevalence as 2–8%, and high prevalence as >8% HBsAg positivity. Findings from our present study support several scientific articles which posit that HBV is endemic in developing countries [14]. The seroprevalence rate in this study is comparable with rates of 8.2-8.3% reported among apparently healthy pregnant women in Nnewi, Yola, and Zaria [17–19]. Similar seroprevalence rates were also reported in Thailand (8.7%) [20], Brazil (8.5%) [21], Dar es Salaam (8.8%) [22], and India (8.35%) [23]. HBV prevalence in this study is higher than 4.1% reported among apparently healthy adolescents in Abakaliki, Southeastern Nigeria [8], 4.7% among HIV patients in Port-Harcourt, Southern Nigeria [24], and 6.02% among children enrolled in an antiretroviral treatment programme in Uyo, South-southern Nigeria [25]. This slightly higher rate may be as a result of differences in population selection. Okonko et al. [26] averred that prevalence rates of HBsAg among different subjects in Nigeria vary significantly at interstate level, depending on frequency of exposure to risk factors. Cultural practices such as tattooing, ear piercing, tribal marking (scarification), and circumcision are widely practiced in underdeveloped countries including Nigeria, and they impact the burden of HBV infection in different population groups [27]. Therefore, indulging in traditional methods of circumcision whereby unsterilized sharp objects are used is likely to be the reason for higher prevalence in this present study [6].

A previous study reported significant association between patients' age and HBsAg seropositivity in Abeokuta, Nigeria [28]. Our present study supports this report because higher HBsAg prevalence was found among age group 24–44 yrs than in other age groups. Age of peak infection in this study is in consonance with reports from previous studies [27, 28]. This is indicative of the role of sexual intercourse in HBV transmission, being the most sexually active group.

In this study, males were more infected compared to females, although there was no significant association with HBsAg seropositivity (Table 2). This is consistent with reports in Nigeria [29–31] and agrees with reports from Southeastern Turkey [32]. However, it is contrary to findings by other researchers who reported higher prevalence in females [24, 33]. Preponderance of male infection in this study may be as a result of increased level of sexual promiscuity among males than females which was reported in some studies in Nigeria [34, 35]. Higher HBsAg carrier status in males can be attributed to the fact that males are less likely to clear HBsAg compared to females [35–37].

Marital status was significantly associated with HBsAg seropositivity in this study. This finding is similar to previous reports in Plateau State [38], Anambra State [39], and Kano State [29]. Although, HBsAg status of patients' spouses is not known in this study, it may not be unconnected with sexual exposures which have been cited in previous studies [30, 31].

Occupational distribution of HBsAg shows that businessmen have higher prevalence of HBV compared to other occupational groups. This is similar to previous reports [17, 26, 29, 40]. History of blood transfusion is the most outstanding risk factor in the present study. In 2007 and 2011, similar reports were made in Ankpa and Anyigba in Kogi State [30, 41], which shows that HBV infection is unabated and hence the need for routine screening and vaccination to interrupt transmission. Exposure to unscreened blood and occult HBV infection are plausible reasons for higher HBsAg seropositivity among previously transfused patients. Although blood transfusion saves millions of lives each year, in developing countries, recipients are at risk of becoming infected with bloodborne diseases such as HBV when blood and/or blood products are not screened before administration [42]. This high prevalence of HBsAg seropositivity in relation to blood transfusion in Ankpa (Table 1) is worrisome and reinforces the need to strengthen state and national policies on blood transfusion, with a view to curtailing HBV transmission through this route.

In this present study, patients with a history of surgeries were 3 times more likely to have HBsAg seropositivity. This positive correlation is similar to reports in Benue State, Nigeria, and Ethiopia [43, 44]. Patients who shared sharp objects had 11.62 times chance of contracting HBV infection. This must have resulted from wounds during exchange or reuse of sharp instruments. Contact of nonintact skin or oral mucosa with secretions or saliva containing HBV from an HBV-infected partner/family member can lead to transmission [45]. This is a likely reason for the significant association of mouth-to-mouth kissing and HBV infection. In this study, patients with HBV patients at home were 4.14 times more likely to contract HBV infection (Table 1). This is in conformity with reports among antenatal clinic women in Yaounde, Cameroon, where history of kissing and contact with jaundiced or HBV-infected person were identified as significant predictors of HBsAg positivity [46]. Strong association observed between alcohol consumption and HBV infection (Table 1) is similar to a previous report by Ndako et al., [9] but in contrast to findings by Mbaawuaga et al. [43]. Patients who indulge in multiple sexual behaviors had about 3.39 times chance of contracting HBV infection. This is justified in previous reports among sexually transmitted and bloodborne infections, where high-risk individuals were reported to have higher probability of getting infected with HBV due to its low infectious dose [47]. The significant association of having multiple sexual partners with HBsAg seropositivity lends credence to the role of sexual intercourse in HBV transmission as highlighted in previous studies [7, 9, 29].

In conclusion, this study highlights endemicity of HBV in Ankpa, Kogi State. Infection is highest among patients within the age group 24–44 yrs which correlate with the age of highest sexual activity. A high rate of HBV among febrile patients in this study informs the need for routine screening of asymptomatic people especially those in the sexually active group to enable early detection and intervention. We recommend mass immunization against HBV and public health education to enlighten people of the observable risk factors and routes of infection. Furthermore, a wider study involving rural dwellers and urban distribution is needed to determine burden of the disease.

## Figures and Tables

**Table 1:** HBV infection and potential risk factors of transmission.

Risk	No. tested	No. positive	OR	CI	*p* value
*1. Knowledge of HBV infection*					
Yes	42	3 (7.14)	0.79	0.22–2.89	0.72
No	158	14 (8.86)			

*2. Mouth-to-mouth kissing*					
Yes	128	17 (13.28)	0.00	0.00	0.001
No	72	0 (0.00)			

*3. Sharing of sharp objects*					
Yes	53	13 (24.53)	11.62	3.59–37.59	0.01
No	147	4 (2.72)			

*4. Multiple sexual partners*					
Yes	46	8 (17.39)	3.39	1.23–9.38	0.014
No	154	9 (5.84)			

*5. History of STDs*					
Yes	34	5 (14.71)	2.21	0.73–6.76	0.15
No	166	12 (7.23)			

*6. Use of condom*					
Yes	104	10 (9.62)	1.35	0.49–3.71	0.56
No	96	7 (7.29)			

*7. History of blood transfusion*					
Yes	48	13 (27.08)	13.74	4.22–44.71	0.01
No	152	4 (2.63)			

*8. History of surgery*					
Yes	34	6 (17.65)	3.02	1.03–8.83	0.04
No	166	11 (6.63)			

*9. Immunized for HBV*					
Yes	12	2 (16.67)	2.31	0.46–11.51	0.30
No	188	15 (7.98)			

*10. History of IDU*					
Yes	17	1 (5.89)	0.65	0.08–5.25	0.69
No	183	16 (8.74)			

*11. Tribal marks*					
Yes	62	5 (8.06)	0.92	0.31–2.74	0.88
No	138	12 (8.70)			

*12. Family history of HBV infection*					
Yes	12	3 (23.08)	4.14	1.01–17.06	0.04
No	188	14 (7.45)			

*13. Alcoholism*					
Yes	59	12 (20.33)	6.94	2.32–20.75	0.01
No	141	5 (3.55)			

**Table 2:** Distribution of HBsAg in relation to patients' sociodemographic characteristics.

Variable	No. tested	No. positive (%)	*p* value
*Age range*			
3–23	74	1 (1.35)	0.01
24–44	91	14 (15.38)	
45–65	25	2 (8.00)	
66–86	10	0 (0.00)	

*Gender*			
Male	101	11 (10.89)	0.22
Female	99	6 (6.06)	

*Marital status*			
Single	97	4 (4.12)	0.03
Married	103	13 (12.62)	
Divorced	0	0 (0.00)	

*Occupation*			
Business men/women	28	8 (28.57)	0.01
Students	95	2 (2.11)	
Housewives	34	5 (14.71)	
Civil servants	29	1 (3.45)	
Farmers	7	1 (14.29)	
Unemployed	7	0 (0.00)	

## Data Availability

The data used to support the findings of this study are included within the article.

## References

[1] Karimi-Googheri M., Daneshvar H., Nosratabadi R. (2014). Important roles played by TGF-*β* in hepatitis B infection. *Journal of Medical Virology*.

[2] World Health Organization (WHO) (2013). *Fact Sheet Number 204: Hepatitis B*.

[3] World Health Organization (WHO) (2013). *Hepatitis B. World Health Organization*.

[4] Williams R. (2006). Global challenges in liver disease. *Hepatology*.

[5] World Health Organization (WHO) (2010). *Prevalence of Hepatitis Virus Infection in The World by Country*.

[6] Odaibo G. N., Arotiba J. T., Fasola A. O., Obiechina A. E., Olaleye O. D., Ajagbe H. A. (2003). Prevalence of hepatitis B virus surface antigen (HBsAg) in patients undergoing extraction at the University College Hospital, Ibadan. *African Journal of Medical and Health Sciences*.

[7] Uneke C., Ogbu O., Inyama P., Anyanwu G., Njoku M., Idoko J. (2005). Prevalence of hepatitis-B surface antigen among blood donors and human immunodeficiency virus-infected patients in Jos, Nigeria. *Memórias Do Instituto Oswaldo Cruz*.

[8] Ugwuja E., Ugwu N. (2010). Seroprevalence of hepatitis B surface antigen and liver function tests among adolescents in Abakaliki, South eastern Nigeria. *International Journal of Tropical Medicine*.

[9] Ndako J. A., Echeonwu G. O. N., Nwankiti O. O. (2012). Hepatitis B virus sero-prevalence among pregnant females in northern Nigeria. *Research Journal of Medical Sciences*.

[10] Sadoh A. E., Sadoh W. E. (2013). Serological markers of hepatitis B infection in infants presenting for their first immunization, Niger. *Journal of Paediatrics*.

[11] Musa B. M., Samaila A. A., Borodo M. M., Femi O. L., Borodo M. M., Bussell S. (2015). Prevalence of hepatitis B virus infection in Nigeria, 2000-2013: a systematic review and meta-analysis. *Nigerian Journal of Clinical Practice*.

[12] Bussell O. M., Wahab D. A. A. A., Adekanle T. S., Okoh A. I. (2012). Seroprevalence of hepatitis B surface antigenemia and its effects on hematological parameters in pregnant women in Osogbo, Nigeria. *Virology Journal*.

[13] Pantazis K. D., Elefsiniotis I. S., Papaioannou D., Brokalaki H., Bonatsos G., Mavrogiannis C. (2008). Large intestine histopathology of pegylated-interferon-alpha plus ribavirin treated chronic hepatitis C patients. *Gastroenterology Research and Practice*.

[14] Zampino R., Boemio A., Sagnelli C (2015). Hepatitis B virus burden in developing countries. *World Journal of Gastroenterology*.

[15] Libbus M. K., Phillips L. M. (2009). Public health management of perinatal hepatitis B virus. *Public Health Nursing*.

[16] Nguyen V. T.-T., McLaws M.-L., Dore G. J. (2007). Highly endemic hepatitis B infection in rural Vietnam. *Journal of Gastroenterology and Hepatology*.

[17] Eke A. C., Eke A. U., Okafor C. I., Ezebialu I. U., Ogbuagu C. (2011). Prevalence, correlates and pattern of hepatitis B surface antigen in a low resource setting. *Virology Journal*.

[18] Olokoba A., Salawu F., Danburam A. (2011). Hepatitis B virus infection amongst pregnant women in North-Eastern Nigeria- A call for action. *Nigerian Journal of Clinical Practice*.

[19] Luka S. A., Ibrahim M. B., Iliya S. (2008). Seroprevalence of Hepatitis B surface antigen among pregnant women attending antenatal clinic in Ahmadu Bello University Teaching Hospital Zaria. *Nigerian J. of Paras*.

[20] SungKanuparph S., Vihagool A., Manosathi W., Kiertiburanakul S., Atamasirikul K. (2004). Prevalence of hepatitis B virus and hepatitis C virus Co-infection with human immunodeficiency virus in Thai patients: a tertiary-based study. *Journal of the Medical Association of Thailand*.

[21] Souza M. G. d., Passos A. D. C., Machado A. A., Figueiredo J. F. d. C., Esmeraldino L. E. (2004). Co-infecção HIV e vírus da hepatite B: prevalência e fatores de risco. *Revista da Sociedade Brasileira de Medicina Tropical*.

[22] Matee M. I., Magesa P. M., Lyamuya E. F. (2006). Seroprevalence of human immunodeficiency strategy to eliminate virus, hepatitis B and C viruses and syphilis infections among blood donors at the Muhimbili National Hospital in Dar es Salaam, Tanzania. *BMC Public Health*.

[23] Pal A., Panigrahi R., Biswas A. (2012). Influence of HIV associated degree of immune suppression on molecular heterogeneity of HBV among HIV coinfected patients. *Virology*.

[24] Frank-Peterside N., Ayodele M. B. O. (2016). Sero-prevalence of hepatitis B virus infection among HIV Co-infected patients in Port harcourt, Nigeria. *New York Science Journal*.

[25] Ikpeme E. E., Etukudo O. M., Ekrikpo U. E. (2013). Seroprevalence of HBV and HIV co-infection in children and outcomes following highly active antiretroviral therapy (HAART) in Uyo, South-South Nigeria. *African Health Sciences*.

[26] Okonko I. O., Soleye F. A., Amusan T. A. (2010). Seroprevalence of HBsAg among patients in Abeokuta, South western Nigeria. *Global Journal of Medical Research*.

[27] Elduma A. H., Elgabar H. E. A. (2015). Coinfection of hepatitis B and C in patients infected with HIV in Sudan. *American Journal of Research Communication*.

[28] Ojo D. A., Ogwu R. S. A., Okerentugba P. O., Okonko I. O. (2013). Prevalence of hepatitis B virus (HBV) seropositivity in a cohort of people living with HIV and AIDS in Abeokuta, ogun state, southwestern Nigeria. *Natural Science*.

[29] Mohammed Y., Sharif A., Dabo N. (2015). Seroprevalence of HBsAg among patients with febrile illnessess in murtala muhammad specialist hospital, Kano, Nigeria. *Bayero Journal of Pure and Applied Sciences*.

[30] Sule W. F., Okonko I. O., Yunusa I. P., Odu N. N., Frank-Peterside N. (2011). Hepatitis B surface antigen (HBsAg) and risk factors of transmission among patients attending hospital in Ankpa, Kogi State, Nigeria. *Nature and Science*.

[31] Udeze A. O., Aliyu A. S., Kolawole O. M., Okonko I. O., Sule W. F., Akanbi K. (2012). Hepatitis B surface antigenaemia and risk factors of transmission among apparently healthy students of university of ilorin, ilorin-Nigeria. *Scientia Africana*.

[32] Mehmet D., Meliksah E., Serif Y., Gunay S., Tuncer O., Zeynep S. (2005). Prevalence of Hepatitis B infection in the southeastern region of Turkey: comparison of risk factors for HBV infection in rural and urban areas. *The Journal of Infectious Diseases*.

[33] Babalola E. T., Ainabe O. B., Okonko I. O. (2013). Confirmation of hepatitis B surface antigen (HBsAg) among selected tertiary institution students in ogun state, Nigeria. *Natural Science*.

[34] UNSN, Nigerian Common Country Assessment (2001). *United Nations Systems in Nigeria*.

[35] Lawal O. A., Bakarey A. S., Uche L. N., Udeze A. O., Okonko I. O. (2009). Hepatitis B virus infection among prospective human immunodeficiency virus positive blood donors at two designated blood banks in ibadan, Nigeria. *World Applied Science Journal*.

[36] Ola S. O., Odaibo G. N., Olaleye D. O. (2004). HCV and HBV infections in Nigerian patients with liver cirrhosis and hepatocellular carcinoma. *Nigerian Quarterly Journal of Hospital Medicine*.

[37] Okonko I. O., Okerentugba P. O., Akinpelu A. O. (2012). Prevalence of HBsAG among attendees of reproductive family and health (ARFH) center in ibadan, southwestern Nigeria. *American-Eurasian Journal of Scientific Research*.

[38] Sirisena N. D., Njoku M. O., Idoko J. A. (2002). Hepatitis B surface antigenaemia in patients with human immunodeficiency virus-1 (HIV-1. Infection in jos, Nigeria. *Nigerian Medical Practitioner*.

[39] Ezegbudo C. N., Agbonlahor D. E., Nwobu G. O. (2004). The seroprevalence of hepatitis B surface antigen and human immunodeficiency virus among pregnant women in Anambra state, Nigeria. *Shiraz E-Medical Journal*.

[40] Amuta E. U., Houmsow R. S., Sar T. T., Awodi E. M. (2012). Seroprevalence of hepatitis B among hospital patients in Makurdi metropolis, Benue State, Nigeria. *International Journal of Biology, Pharmacy and Allied Sciences*.

[41] Sule W. F., Abraham-Oyiguh J., Abdultalib Y., Taiga A., Abba M. A. (2007). Seroprevalence of hepatitis B surface antigen (HBsAg) among pregnant women in Anyigba, Kogi State, Nigeria. *Journal of Applied Science and Environmental Studies*.

[42] UNAIDS Women and AIDS (2007). *UNAIDS Point of View*.

[43] Mbaawuaga E. M., Christian U. I., Anthony C. I., Godwin T. A. J. (2014). Studies on prevalence, co-infection and associated risk Factors of hepatitis B virus (HBV) and human Immunodeficiency virus (HIV) in Benue State, Nigeria. *Science Journal of Public Health*.

[44] Zenebe Y., Mulu W., Yimer M., Abera B. (2014). Sero-prevalence and risk factors of hepatitis B virus and human immunodeficiency virus infection among pregnant women in Bahir Dar city, Northwest Ethiopia: a cross sectional study. *BMC Infectious Diseases*.

[45] Idea S. Hepatitis B-The Facts: Victorian Government Health Information, Australia, State of Victoria

[46] Fomulu N. J., Morfaw F. L., Torimiro J. N., Nana P., Koh M. V., William T. (2013). Prevalence, correlates and pattern of Hepatitis B among antenatal clinic attenders in Yaounde-Cameroon: is perinatal transmission of HBV neglected in Cameroon?. *BMC Pregnancy Childbirth*.

[47] Chang M.-H. (2007). Hepatitis B virus infection. *Seminars in Fetal and Neonatal Medicine*.

